# Comparative study of quality of life of adult survivors of childhood acute lymphocytic leukemia and Wilms’ tumor

**DOI:** 10.1590/S1679-45082015AO3231

**Published:** 2015

**Authors:** Clélia Marta Casellato de Souza, Lilian Maria Cristofani, Ana Lucia Beltrati Cornacchioni, Vicente Odone, Evelyn Kuczynski

**Affiliations:** 1 Faculdade de Medicina, Universidade de São Paulo, São Paulo, SP, Brazil.; 2 Hospital das Clínicas, Faculdade de Medicina, Universidade de São Paulo, São Paulo, SP, Brazil.; 3 Instituto da Criança, Hospital das Clínicas, Faculdade de Medicina, Universidade de São Paulo, São Paulo, SP, Brazil.

**Keywords:** Quality of life, Leukemia, Wilms tumor, Survivors, Questionnaires

## Abstract

**Objective:**

To analyze and compare the health-related quality of life of adult survivors of acute lymphocytic leukemia and Wilms’ tumor amongst themselves and in relation to healthy participants.

**Methods:**

Ninety participants aged above 18 years were selected and divided into three groups, each comprising 30 individuals. The Control Group was composed of physically healthy subjects, with no cancer history; and there were two experimental groups: those diagnosed as acute lymphocytic leukemia, and those as Wilms’ Tumor. Quality of life was assessed over the telephone, using the Medical Outcomes Study 36-Item Short Form Health Survey.

**Results:**

Male survivors presented with better results as compared to female survivors and controls in the Vitality domain, for acute lymphocytic leukemia (p=0.042) and Wilms’ tumor (p=0.013). For acute lymphocytic leukemia survivors, in Social aspects (p=0.031), Mental health (p=0.041), and Emotional aspects (p=0.040), the latter also for survivors of Wilms’ tumor (p=0.040). The best results related to the Functional capacity domain were recorded for the experimental group that had a late diagnosis of acute lymphocytic leukemia. There were significant differences between groups except for the Social and Emotional domains for self-perceived health, with positive responses that characterized their health as good, very good, and excellent.

**Conclusion:**

Survivors of acute lymphocytic leukemia showed no evidence of relevant impairment of health-related quality of life. The Medical Outcomes Study 36-Item Short Form Health Survey (via telephone) can be a resource to access and evaluate survivors.

## INTRODUCTION

Worldwide incidence of childhood cancer is estimated between 1 and 3% of the total number of cases of the disease in most populations. According to data from the Population-Based Cancer Registry, Brazil is close to 3%, with relevant incidence rates of acute lymphocytic leukemia (ALL) and of Wilms’ tumor (WT), among other tumors.^(1,2)^

With the progress of treatment, worldwide mortality of children with cancer (particularly leukemias, lymphomas, and solid tumors) has shown a significant drop since the 1960’s.^(3)^

Therefore, over the last decades, the approach of disease has focused on knowledge of its late effects and choices of treatment, to identify the risk factors to physical and psychosocial health of the adult survivor of childhood cancer. These parameters allow a more effective follow-up of long-term survival demands on the part of the multidisciplinary team.^(4)^ Such knowledge can be obtained by means of instruments (questionnaires) that evaluate health-related quality of life (HRQoL).^(5,6)^

Particularly in this population, there is evidence of post-traumatic stress situations and reluctance of the survivor to return to the place of treatment associated with the traumatizing condition.^(7)^

In a recent study, the importance of including on-line services for support follow-up in cancer survivors has been highlighted.^(8)^

In this sense, by means of the Medical Outcomes Study 36-Item Short Form Health Survey (SF-36) questionnaire, we aimed to evaluate the HRQoL of adult survivors of childhood ALL and TW followed up at an outpatient clinic of an institute specialized in treating childhood cancer. An alternative form of application (via telephone) of the instrument, which is already established and recognized in the scientific world, was used to assess the HRQoL of patients and healthy subjects.

Survivors of ALL and WT, as occurs in outpatient treatments to control other chronic diseases, have periodic contacts (annual or even at long intervals) with the multidisciplinary team, for control/monitoring tests during the period off oncologic treatment.

The proposal of a distant evaluation as one more resource to improve the follow-up process arose from this particularity of the population studied.

The interest of this study in comparatively analyzing the HRQoL of ALL and WT survivors amongst themselves and relative to healthy individuals derived from the different treatments used for remission of the neoplasm.

In ALL, a malignant disease of the hematopoietic system, there are frequently more drastic treatments and more prolonged hospitalizations. Nevertheless, the survivor generally does not present with marks on the body of the episode that occurred during childhood. On the other hand, in nephroblastoma or WT, there has probably been surgical treatment, which leaves a scar.

It is important to point out that there are few studies on HRQoL comparing ALL and WT survivors.^(9-11)^ and the relevance of studies that focus, analyze, and compare certain survival conditions.

## OBJECTIVE

To analyze and compare the health-related quality of life of adult survivors of childhood acute lymphocytic leukemia and Wilms’ tumor among themselves and relative to healthy participants.

## METHODS

This project was approved by the Ethics Committee for Analysis of Research Projects (CAPPesq) of the Clinical Board of the *Hospital das Clínicas da Faculdade de Medicina da Universidade de São Paulo*, (HC-FMUSP) protocol number 0458/11, on July 29, 2011.

The study had a Control Group (CG) of healthy participants and two experimental groups of survivors.

The CG was composed of 30 participants (15 men and 15 women), aged over 18 years and selected from the general population. Only physically healthy participants were included, with no history of oncologic diagnosis at any time of life and recently enrolled in a higher education course. With these criteria, we aimed to establish a convenience sample that allowed a comparison with individuals who basically were physically healthy and had a good cultural level.

For the experimental groups, the survivors selected were aged over 18 years, of both sexes, who had been followed up for at least five years at the Out-of-Treatment Outpatient Clinic of the *Instituto de Tratamento do Câncer Infantil (*ITACI*) *of the* Serviço de Onco-Hematologia Pediátrica do* HC-FMUSP. The individuals were divided into one experimental group composed of 30 ALL survivors (ALLG) and one experimental group composed of 30 WT survivors (WTG).

Data collection of all groups was performed by means of the application, via telephone contact, of the SF-36 questionnaire, which was validated for the Portuguese language by physical presence.^(12)^ In a posterior viability analysis of an alternative form of application (via telephone contact), the equality of the two forms of presentation – in person and by telephone - was detected.^(13)^ The mean time of application of the questionnaire (by telephone) was 12.3 minutes. The data were collected from September 2011 to August 2013.

Each participant was previously explained the purposes, procedure, and degree of risk of the research project to their health. Based on their agreement, the Informed Consent Form was signed for participation in the project.

With the objective of enriching the study and establishing possible relevant correlations in the results obtained for the SF-36 domains, in addition to collection of responses to the questions that make up the instrument used, some updated clinical and sociodemographic data were selected by means of consultation of the patient’s medical records (age at time of diagnosis and time out of therapy) or with the participant, during the telephone contact, before applying the SF-36 (marital status, children, level of schooling, professional occupation, average monthly gross family income).

The data obtained through application of the SF-36 questionnaire were compiled according to the validation criterion for the Portuguese language.^(12)^ Gross family income of the participants was grouped into two minimum wage ranges (less than or equal to 2.5 minimum monthly wages and above 2.5 minimum monthly wages), considering as reference the values obtained in the socioeconomic survey of 2011, by the *Instituto Brasileiro de Opinião Pública e Estatística* (IBOPE).^(14)^

Statistical analysis of the results was done using the Statistical Package for the Social Sciences (SPSS) software, by means of χ^2^ and independent *t* tests, and variance analysis (ANOVA).

The significance level was considered as p=0.05, except one analysis in which the significance of p=0.10 was checked, because, in this case, significance was evident on ANOVA (p=0.05). However, this difference in least squares multiple comparison (LSMC) was only detected for p=0.10.

## RESULTS

On table 1, the sociodemographic characteristics of the experimental and CGs are presented.

**Table 1:** Sociodemographic profile of the groups of participants

Sociodemographic characteristics		Groups			p value
		ALLG	WTG	CG	
		n (%)	n (%)	n (%)	
Age (years)	18-25	14 (46.6)	18 (60.0)	23 (76.6)	0.058^#^
	≥26	16 (53.4)	12 (40.0)	7 (23.4)	
Sex	Female	15 (50.0)	15 (50.0)	15 (50.0)	
	Male	15 (50.0)	15 (50.0)	15 (50.0)	
Marital status	Single	25 (83.3)	21 (70.0)	27 (90.0)	0.131^#^
	Married or in a stable union/divorced/separated	5 (16.7)	9 (30.0)	3 (10.0)	
Children*	Yes	3 (10.0)	7 (23.4)	2 (6.6)	^##^
	No	27 (90.0)	23 (76.6)	28 (93.4)	
Schooling	Incomplete Elementary up to Complete High School	15 (50.0)	13 (43.3)	0 (0.0)	<0.001^### ^
	Incomplete Undergraduate	7 (23.4)	7 (23.4)	22 (73.4)	
	Complete Undergraduate and Graduate	8 (26.6)	10 (33.4)	8 (26.6)	
Situation in the job market	Employed				^##^
	Managerial positions (supervisors, managers, etc.)	4 (13.3)	6 (20.0)		
	Other hierarchical levels	16 (53.4)	16 (53.4)	19 (63.4)	
	Self-employed	3 (10.0)	3 (10.0)	4 (13.3)	
	Unemployed	2 (6.6)	1 (3.3)		
	Housework	1 (3.4)	1 (3.3)		
	Student/does not work	4 (13.3)	3 (10.0)	7 (23.3)	
Demographic place of origin (regions)	Southeast				^##^
	São Paulo	29 (96.7)	24 (80.0)	30 (100.0)	
	Minas Gerais	1 (3.3)	4 (13.4)		
	Rio de Janeiro		1 (3.3)		
	South				
	Paraná		1 (3.3)		
Gross [monthly] family income (MW)	≤2.5^$^	12 (40.0)	9 (30.0)		^##^
	>2.5^$$^	18 (60.0)	21 (70.0)	30 (100.0)	

^#^ χ^2^ (p=0.05); ^##^χ^2^: unfeasible application (low incidence of some categories); ^###^χ^2^ (p=0.05); comparative by ALLG *versus* WTG partitions (p=0.083); ALLG *versus* CG (p<0,001); WTG *versus* CG (p<0.001); ^$^range of gross [monthly] family income in reference of socioeconomic survey, in 2011, by the IBOPE* Mídia* and the *Associação Brasileira de Estudos Populacionais* (ABEP),^(14)^ that covers classes C and D (*Critério Brasil* - 2013); ^$$^range of gross [monthly] family income in reference to the socioeconomic survey, in 2011, as per IBOPE* Mídia/*ABEP*,* ^(14)^ that covers classes A and B (*Critério Brasil* - 2013). ALLG: experimental group of acute lymphocytic leukemia survivors; WTG: experimental group of Wilms’ tumor [survivors]; CG: Control Group; MW: minimum wages.

There was no evidence of a significant difference as to age of the participants of the three groups studied (χ^2^ test [2]=5.704; p=0.058), with the CG showing the highest concentration of younger participants (76.6% up to 25 years of age) and the ALLG with the highest concentration of older survivors (53.4% over 26 years of age).

Most of the participants are single, with no children, and from São Paulo.

The CG showed a higher level of schooling relative to the experimental groups. This occurred due to the inclusion criterion for the CG (χ^2^ test [4]=27.02; p<0.001). Nevertheless, there was no significant difference between the experimental groups (χ^2^ test [2]=0.365; p=0.833). There was a high concentration of survivors who had finished up to High School (50.0% of ALLG and 43.3% of the WTG) and a considerable proportion of survivors with incomplete Higher Education (23.4% of the ALLG and 23.4% of the WTG), complete Higher Education and graduate (26.6% of the ALLG and 33.4% of the WTG).

As to occupation, most the participants from all the groups were employees from various hierarchical levels, including leadership positions. The mean family [monthly] income was over 2.5 minimum wages (Classes B and A as per IBOPE-ABEP).^(14)^

Table 2 shows the clinical characteristics of the experimental group participants.

**Table 2:** Clinical characteristics of the experimental groups

Clinical characteristics	ALLG	WTG	p value
	Mean (SD)	Mean (SD)	
Age at diagnosis (months)	57.6 (37.2)	45.4 (25.8)	0.147
Treatment period (months)	39.6 (18.0)	12.7 (7.9)	<0.001
Period off treatment (years)	17.6 (4.7)	19.7 (4.6)	0.090

ALLG: experimental group of acute lymphocytic leukemia survivors; WTG: experimental group of Wilms’ tumor survivors; SD: standard deviation.

As to the therapeutic history, there were no significant differences as to age at the time of diagnosis (independent *t* test, *t *(58) =1.472; p=0.147) and the period of time off treatment for the survivors analyzed (independent *t* test, *t* (56) =-1.725; p=0.090). In the WTG, however, there was a tendency towards a greater proportion of patients diagnosed at a young age range, with 60% of them having been diagnosed before 52 months of age (mean of 45.4 months; standard deviation − SD 25.8), and although with no significant difference, there was also in this group a greater proportion of survivors off treatment for a longer time: 64.3% of the survivors for more than 20 years (mean 19.7 months; SD 4.6). We note that the treatment given to the ALLG patients was, on average, significantly (independent *t* test, *t* (58) =-7.37, p<0.001*) more prolonged (mean 39.6 months; SD 18.0).

Table 3 shows a summary of the significant results obtained in the statistical analysis for the SF-36 domains relative to age, sex, and age at time of diagnosis for the study participants.

**Table 3:** Statistical analysis of the Medical Outcomes Study 36-Item Short Form Health Survey domains relative to factors age, sex, and age at diagnosis

Factors/groups	Mean	SD	ANOVA	LSMC
			(p value)	(p value)
**Age **	**Functional capacity (≤25/>26 years)**			
ALLG	96.43/92.19	4.97/15.92		
WTG	93.61/90.42	7.24/10.33	0.038	
CG	94.78/85.71	10.92/20.90		0.072
**Sex**	**Vitality (female/male)**			
ALLG	60.67/75.00	29.27/16.26		0.042
WTG	58.33/76.00	17.80/16.28	0.010	0.013
CG	63.00/63.00	19.35/11.77		
**Social aspects (female/male)**				
ALLG	77.50/95.83	29.58/6.10		0.031
WTG	76.67/85.83	28.29/23.08	0.024	
CG	76.67/82.50	20.52/22.06		
**Emotional aspects (female/male)**				
ALLG	55.56/86.67	46.58/24.56		0.040*
WTG	55.56/71.11	46.58/45.19	0.038	0.040*
CG	82.22/60.00	33.01/44.01		
**Mental health (female/male)**				
ALLG	63.47/79.47	23.07/16.20		0.012
WTG	65.60/73.60	19.64/18.50	0.028	
CG	73.87/74.13	11.80/10.57		
**Age at diagnosis**	**Functional capacity (≤52/>53 months)**			
ALLG	90.94/97.86	15.08/4.69		0.041**
WTG	94.17/89.58	6.70/10.54	0.033	

*ALLG male/WTG female; **ALLG >53/WTG >53; SD: standard deviation; ANOVA: variance analysis; LSMC: least squares multiple comparison; ALLG: experimental group of acute lymphocytic leukemia survivors; WTG: experimental group of Wilms’ tumor survivors; CG: Control Group.

As to the age of the participants, a significant difference was evident [ANOVA, (F [1.84]=4.438; p=0.038*)] for the Functional capacity domain. This difference was only detected when the level of significance (p) 0.10 was adopted; the younger participants of the CG presented with greater responses for this domain (p=0.072*).

There was also a significant difference [ANOVA, (F [1.56]=4.780; p=0.033*)] in the Functional capacity domain when considering the age factor at the time of diagnosis. Among the survivors who had a later diagnosis (over 53 months of age), those with ALLG had significantly greater values of responses (p=0.041*).

As to sex, significant differences were found only among the survivors, specifically for the Vitality domains [ANOVA, (F [1.84]=6.933; p=0.010*)], Social aspects [ANOVA, (F [1.84]=5.286; p=0.024*)], Emotional aspects [ANOVA, (F [2.84]=3.382; p=0.038*)], and Mental health [ANOVA, (F [1.84] =4.980; p=0.028*)]. The male survivors of groups ALLG (p=0.044) and WTG (p=0.013) had greater values for the responses to the Vitality domain. The male survivors of ALLG (p=0.031) showed greater values for the responses of the Social aspects domain. Among the participants of a same group, only in ALLG (p=0.040) did the survivors present with greater results for the Emotional aspects domain. However, for participants of different groups, it was noted that the ALLG survivors had higher responses (p=0.040) for the Emotional aspects domain when compared to those of the WTG. ALLG survivors presented with higher values for the responses to the Mental health domain (p=0.012*).

Table 4 shows the perception of the participants of their own health (question 1 of SF-36, where the possible answers are good, very good, and excellent) relative to the SF-36 domains.

**Table 4:** Statistical analysis of the factor perception of one’s own health by the Medical Outcomes Study 36-Item Short Form Health Survey

Factors/groups	Mean	SD	ANOVA (p value)	LSMC (p value)
**Perception of health**	**Functional capacity (good/very good/excellent)**			

ALLG	85.00/95.00/98.67	19.46/7.07/2.97		0.006^§^; 0.025^§§^; 0.021^§§§^
WTG	87.73/92.50/96.82	9.84/8.02/5.13	0.002	0.015^£^; 0.048^££^;
CG	90.36/91.67/98.57	15.75/16.20/2.44		0.048^£££^
**Physical aspects (good/very good/excellent)**				
ALLG	78.13/100.0/100.0	33.91/0.00/0.00		0.019^#^
WTG	84.09/87.50/97.73	35.83/26.73/7.54	0.0250	
CG	78.57/69.44/96.43	30.79/41.04/9.45		0.026^##^; 0.005^###^; 0.015^#$^; 0.039^#$$^
**Pain (good/very good/excellent)**				
ALLG	63.00/75.57/83.13	24.09/19.60/18.32		0.024^&^;0.021^&&^; 0.012^&&&^
WTG	65.18/70.38/84.73	16.98/22.80/20.16	0.0021	0.026^¢^; 0.024^¢¢^; 0.014^¢¢¢^
CG	73.29/79.00/89.43	20.48/24.14/10.50		
**General health status (good/very good/excellent)**				
ALLG	67.63/81.57/94.33	25.56/15.08/8.42		0.0004q; 0.003qq; 0.003qqq
WTG	62.45/72.00/90.45	21.03/12.82/11.28	<0.0001	0.018w; 0.014e; <0.0001r; 0.0001t; 0.0002y; 0.002p; 0.018l; 0.013k
CG	57.00/80.89/93.57	24.02/7.41/7.48		0.001j; 0.043h; 0.001g; <0.0001d; 0.0001s; 0.0001a
**Vitality (good/very good/excellent)**				
ALLG	46.25/65.00/80.67	27.48/14.72/17.71		0.014z; 0.011c; <0.0001v; 0.006b
WTG	62.73/69.38/70.00	24.12/12.94/17.75	0.0032	
CG	58.21/69.44/64.29	18.87/11.58/11.34		0.001n
**Mental health (good/very good/excellent)**				
ALLG	56.50/66.29/81.87	27.91/18.60/11.70		0.046m; 0.023aa; 0.001^€^; 0.0247∞; 0.047Ω
WTG	66.18/72.50/70.91	21.34/14.88/20.87	0.0305	0.022^¶^
CG	71.71/75.56/76.57	11.26/11.39/10.69		

^§^good ALLG/excellent ALLG; ^§§^good ALLG/excellent WTG; ^§§§^good ALLG/excellent CG;^ £^good WTG/excellent ALLG; ^££^good WTG/excellent CG; ^£££^good CG/excellent ALLG; ^#^very good ALLG/very good CG; ^##^good CG/excellent ALLG; ^###^very good CG/excellent ALLG; ^#$^very good CG/excellent WTG; ^#$$^very good CG/excellent CG; ^&^good ALLG/excellent ALLG; ^&&^good ALLG/excellent WTG; ^&&&^good ALLG/excellent CG; ^¢^good WTG/excellent ALLG; ^¢¢^good WTG/excellent WTG; ^¢¢¢^good WTG/excellent CG; qgood ALLG/excellent ALLG; qqgood ALLG/excellent WTG; qqqgood ALLG/excellent CG; wgood WTG/very good ALLG; egood WTG/very good CG; rgood WTG/excellent ALLG; tgood WTG/excellent WTG; ygood WTG/excellent CG; pvery good WTG/excellent ALLG; lvery good WTG/excellent WTG; kvery good WTG/excellent CG; jgood CG/very good ALLG; hgood CG/very good WTG; ggood CG/ very good CG; dgood CG/excellent ALLG; sgood CG/excellent WTG; agood CG/excellent CG; zgood ALLG/very good WTG; cgood ALLG/very good CG; vgood ALLG/excellent ALLG; bgood ALLG/excellent WTG; ngood CG/excellent ALLG; mgood ALLG/good CG; aagood ALLG/excellent ALLG; ^€^good ALLG/very good CG; ∞good ALLG/excellent CG; Ωvery good ALLG/excellent ALLG; ^¶^good WTG/excellent ALLG. SD: standard deviation; ANOVA: variance analysis; LSMC: least squares multiple comparison; ALLG: experimental group of acute lymphocytic leukemia survivors; WTG: experimental group of Wilms’ tumor survivors; CG: Control Group.

A statistical difference was observed for all the groups as to perception of one’s own health for the domains analyzed by the SF-36, except for Social aspects and Emotional aspects. We noted that the significant differences found were sometimes established internally, among participants of the same group, but also among participants of different groups. The complete list of significant relations was shown on the legend of table 4.

## DISCUSSION

To be affected by cancer and survive after remission can be experienced with pain and suffering by patients and their family members. In treatment analyses, it is noteworthy that the advances achieved in treatment and the efforts for improvement of the HRQoL do not avoid disruptive situations established by the impact of the diagnosis, by treatment, and by its implications.^(15)^ According to this perspective of HRQoL compromise of survival, this study found results that need to be discussed.

As to the perception of one’s own health, there were significant differences for all the domains, except for the Social aspects and Emotional aspects. However, we noted that the differences found for ALLG, WTG, and CG were established around the perceptions of good, very good, and excellent health, demonstrating that not all participants seem to denote negative perception of their own health.

In the experimental groups of survivors, age showed no influence in the results for all the SF-36 domains and therefore, neither did it in the HRQoL of these survivors.

Several studies have dealt with the topic of ALL survival and its possible involvement. The work conducted by Robison,^(16)^ Ortolan,^(17)^ and Zebrack et al.^(18)^ is relevant for analysis of the results obtained by this study. The investigation carried out by Robison^(16)^ covered ALL survival in a cohort study. Despite not establishing an analysis of HRQoL, the study analyzed the late effects on ALL survival, highlighting the psychic and social impairment in the survivors of both sexes – a condition not observed in the present study. The best scores of certain aspects of HRQoL of the male survivors in comparison with the ALL survivors obtained in this study, corroborate the results previously disclosed by Ortolan,^(17)^ specifically for the domains of Vitality, Social aspects, and Mental health. Zebrack et al.^(18)^ analyzed particularly the future psychosocial function of the ALL survivor, and found that the survivors were more likely to report aspects of depressive and somatic symptoms than did the controls – the women were more likely to indicate symptoms related to somatic depression and anguish than did the men. In the sample analyzed by this study, psychic compromises were also evident with significantly smaller differences in the Mental health and Emotional aspects domains of SF-36 of the ALLG survivors.

It is important to point out that, despite the differences described above, no mean values below 50 (mean value of the scale) were detected for any of the domains evaluated, in both sexes.

Of the clinical variables analyzed, in the survivors with late diagnoses (aged over 53 months), only the Functional capacity domain showed significantly greater results for the ALLG, diversely obtained by Zebrack et al.,^(18)^ who pointed out that it was the age at the time of diagnosis, the time of diagnosis, and the duration of treatment that were predictive of symptomatic scores for somatic depression or discomfort_._^(18)^

On the other hand, there are few analyses on the HRQoL of the two groups of neoplastic behavior (hematologic and solid, specifically ALL and WT), comparing the charts of survival amongst themselves and particularly for the age range analyzed in this study, such as, for example, Mackie et al.,^(9)^ Stuber and Shemesh.^(10)^

Mackie et al.^(9)^ analyzed psychiatric disorders, interpersonal/social performance, and intellectual capacity of 102 patients (19 to 30 years) who were survivors of childhood ALL and WT and with no relapse during five years comparatively with a CG of healthy subjects. As to the psychiatric disorders, there were no significant differences between the groups analyzed. *Déficits* in the items love relationship/sex and friendship found among the survivors were associated with more recent treatment situations.^(9)^

The study done by Mackie et al.^(9)^ is similar to this study as to age range and neoplastic involvement, but focuses on the analysis of survival specifically as to psychiatric disorders and social and intellectual performance, pointing out *déficits* not detected by this study and not covering other aspects of survival, such as, the physical aspects.

Stuber and Shemesh^(10)^ analyzed the post-traumatic stress disorder in children and adolescents affected by different neoplasms, and in their parents. Despite including young survivors (8 to 20 years), the work by Stuber and Shemesh compared those affected who were studied by this project (309 survivors, 38% of them of ALL and 10% of WT) relative to the healthy CG paired by age. Stuber and Shemesh^(10)^ also did not note any significant difference between the score of symptoms classifiable between serious and moderate for the disorder when compared to the survivor groups and health controls, as was observed in the present study regarding the absence of a significant difference in psychic disorders in survivors relative to controls.

Koch et al.^(11)^ analyzed the HRQoL of survivors and also found no serious compromises in survivors in the same diseases studied by this project relative to the healthy CGs. The authors treated psychosocial aspects by the possibility of leaving the homes of the parents of the survivors. The survivors of hematologic and solid tumors did not significantly differ relative to the CG as to the variable leaving their parents’ homes. They concluded that for the survivor and his/her parents, the type of treatment required (surgery and/or chemotherapy) did not interfere significantly in the psychosocial process in general nor in the person’s having social independence in adulthood.^(11)^ However, when covering the topic of survival of adult patients affected by hematologic cancer and solid tumors, the authors jointly analyzed leukemias/lymphomas and all solid tumors as a group, impeding specificity in the analysis for each disease, a fact that would enable a more rigorous comparison relative to the data obtained in this study.

As to the application of the SF-36 by telephone, as was recommended in this study, Zebrack and Landier^(19)^ pointed out the importance of support follow-up for cancer survivors, including on-line services. Meadows^(7)^ analyzed the detection of post-traumatic stress situations and the reluctance of the survivor to return to the place of treatment, which is associated with the traumatizing condition. In this way, the difference proposed may be considered one more resource to help in the *déficit* of approximation and data collection for clinical follow-up, favoring the gathering of information for scientific investigation, accompaniment of the clinical condition, and intervention, if necessary.^(7,19)^

It is relevant to point out that there are few analyses on health-related quality of life comparing survivors of acute lymphocytic leukemia and Wilms’ tumor. Thus, this study aimed to contribute towards the construction of knowledge on the theme, accumulation of evidence, and the improvement of therapeutic processes.

A greater number of analyses may detect particularities, enriching prior knowledge and providing opportunity for future studies, as was the aim of this research in ALL and WT survivors.

## CONCLUSION

Male survivors had better results relative to the survivors and participants of the Control Group. Specifically, in the experimental groups of survivors of acute lymphocytic leukemia and of Wilms’ tumor for the Vitality domain, and in the experimental group of survivors of acute lymphocytic leukemia for Social aspects, Mental health, and Emotional aspects, where in the latter aspects a difference was also detected relative to the survivors of the experimental group of Wilms’ tumor survivors.

It was only among the survivors with late diagnoses (more than 53 months of age) that there were significant differences in quality of life, in which the experimental group of survivors of acute lymphocytic leukemia presented with better results as to the Functional capacity domain.

As to perception of one’s own health, there was a difference for all domains, except for the Social aspects and Emotional aspects. Nevertheless, the differences found were related to positive perceptions (good, very good, and excellent) of one’s own health in the survivors and healthy controls.

Therefore, for the period of the study and for the sample analyzed, it can be inferred that the survivors analyzed did not present with evidence of compromise of quality of life related to health for the aspects analyzed by the instrument used, as well as when compared to the Control Group of healthy participants.

The results found are feasible for confirmation by future studies, in other participant samples, by other research centers and with other instruments for evaluation of health-related quality of life.

We should also add that the use of the Medical Outcomes Study 36-Item Short Form Health Survey via telephone contacts enabled access and evaluation of the survivors’ quality of life, followed-up as outpatients.
