# Supplementary material for: Comparative study of quality of life of adult survivors of childhood acute lymphocytic leukemia and Wilms’ tumor
Source: Einstein (Sao Paulo). 2015 Oct-Dec;13(4):492–9. doi: 10.1590/S1679-45082015AO3231 (PMC4878620; doi:10.1590/S1679-45082015AO3231)
Supplement: Translation

# Translation: Estudo comparativo da qualidade de vida de adultos sobreviventes de leucemia linfocítica aguda e tumor de Wilms na infância

**Authors:** Clélia Marta Casellato de Souza, Lilian Maria Cristofani, Ana Lucia Beltrati Cornacchioni, Vicente Odone, Evelyn Kuczynski

**Affiliations:** 1 Faculdade de Medicina, Universidade de São Paulo, São Paulo, SP, Brasil.; 2 Hospital das Clínicas, Faculdade de Medicina, Universidade de São Paulo, São Paulo, SP, Brasil.; 3 Instituto da Criança, Hospital das Clínicas, Faculdade de Medicina, Universidade de São Paulo, São Paulo, SP, Brasil.

**Keywords:** Qualidade de vida, Leucemia, Tumor de Wilms, Sobreviventes, Questionários

## Abstract

**Objetivo:**

Analisar e comparar a qualidade de vida relacionada à saúde de sobreviventes adultos de leucemia linfocítica aguda e tumor de Wilms entre si, e em relação a participantes sadios.

**Métodos:**

Foram selecionados noventa participantes, acima de 18 anos, os quais foram divididos em três grupos, sendo cada um com 30 sujeitos: Grupo Controle, que contou com indivíduos fisicamente saudáveis, sem histórico oncológico; grupo experimental formado por pacientes que tiveram diagnóstico de leucemia linfocítica aguda; e grupo experimental formado por pacientes que tiveram diagnóstico de Tumor de Wilms. A avaliação da qualidade de vida foi realizada por telefone e utilizou o *Medical Outcomes Study 36-Item Short Form Health Survey*.

**Resultados:**

Os sobreviventes do sexo masculino apresentaram melhores resultados em relação aos do sexo feminino e controles no Aspecto vitalidade, para leucemia linfocítica aguda (p=0,042) e tumor de Wilms (p=0,013). Para os sobreviventes de leucemia linfocítica aguda nos Aspectos sociais (p=0,031), Saúde mental (p=0,041) e Aspectos emocionais (p=0,040), neste último também para as sobreviventes de Tumor de Wilms (p=0,040). Os melhores resultados relacionados ao domínio Capacidade funcional foram registrados para o grupo experimental de pacientes que tiveram diagnóstico tardio de leucemia linfocítica aguda. Observaram-se diferenças significativas entre os grupos, exceto para os domínios Aspectos sociais e emocionais para a percepção da própria saúde, que teve respostas de cunho positivo, que qualificavam a própria saúde como boa, muito boa e excelente.

**Conclusão:**

O grupo experimental de pacientes que tiveram diagnóstico de leucemia linfocítica aguda não apresentou evidências de comprometimento relevante da qualidade de vida relacionada à saúde. O* Medical Outcomes Study 36-Item Short Form Health Survey* (via telefone) pode ser um recurso de acesso e avaliação de sobreviventes.

## INTRODUÇÃO

A incidência mundial de câncer na infância é estimada entre 1 e 3% do total de casos da doença na maioria das populações. Segundo dados do Registro de Câncer de Base Populacional, o Brasil está próximo dos 3%, com taxas relevantes de incidência de leucemia linfocítica aguda (LLA) e do tumor de Wilms (TW), entre outros tumores.^(1,2)^

Com os progressos da terapêutica, a mortalidade mundial de crianças acometidas por câncer (particularmente leucemias, linfomas e tumores sólidos) apresentou declínio significativo a partir dos anos 1960.^(3)^

Assim, nas últimas décadas, a abordagem da doença pode se voltar ao conhecimento de seus efeitos tardios e dos tratamentos de escolha, para a identificação de fatores de risco à saúde física e psicossocial do adulto sobrevivente do câncer infantil. Esses parâmetros permitem um acompanhamento mais efetivo das demandas da sobrevida de longo prazo por parte da equipe multidisciplinar.^(4)^ Tal conhecimento pode ser obtido por meio de instrumentos (questionários) de avaliação da qualidade de vida relacionada à saúde (QVRS).^(5,6)^

Particularmente nessa população, há evidência de situações de estresse pós- traumático e relutância por parte do sobrevivente de retornar ao local de tratamento, associado à condição traumatizante.^(7)^

Em estudo recente, foi ressaltada a importância da inclusão de serviços *on-line* para o acompanhamento de suporte para sobreviventes de câncer.^(8)^

Portanto, buscou-se, por meio do questionário *Medical Outcomes Study 36-Item Short Form Health Survey* (SF-36), avaliar a QVRS de sobreviventes adultos acometidos por LLA e TW na infância, acompanhados em um ambulatório de instituto especializado no tratamento do câncer infantil. Foi utilizada uma forma de aplicação alternativa (via telefone) do instrumento, já fundamentado e reconhecido no meio científico, para abordagem da QVRS de pacientes e sujeitos saudáveis.

Os sobreviventes de LLA e TW, como ocorrem nos tratamentos ambulatoriais de controle de outras doenças de evolução crônica, têm contatos periódicos (anuais ou até períodos mais longos) com a equipe multidisciplinar, para exames de controle/monitoramento no período fora de terapia oncológica.

A proposta de uma forma de avaliação à distância como mais um recurso de melhoria no processo de acompanhamento de sobreviventes surgiu dessa particularidade da população estudada.

Da diferença dos tratamentos empregados para a remissão da neoplasia surgiu o interesse deste estudo em analisar comparativamente a QVRS de sobreviventes de LLA e TW entre si e em relação aos indivíduos saudáveis.

Na LLA, doença maligna do sistema hematopoiético, têm-se frequentemente tratamentos mais drásticos e internações mais prolongadas. No entanto, o sobrevivente, geralmente, não apresenta marcas no corpo do episódio ocorrido na infância. Por outro lado, no nefroblastoma ou TW, há um possível tratamento cirúrgico, determinante da presença de uma cicatriz.

É importante ressaltar que há poucos estudos sobre a QVRS comparando sobreviventes de LLA e TW,^(9-11)^ e a relevância de estudos que focalizam, analisam e comparam determinados quadros de sobrevivência.

## OBJETIVO

Analisar e comparar a qualidade de vida relacionada à saúde de sobreviventes adultos de leucemia linfocítica aguda e tumor de Wilms entre si e em relação a participantes sadios.

## MÉTODOS

Este trabalho foi aprovado pelo Comitê de Ética para Análise de Projetos de Pesquisa (CAPPesq) da Diretoria Clínica do Hospital das Clínicas da Faculdade de Medicina da Universidade de São Paulo, (HC-FMUSP) protocolo 0458/11, em 29 de julho de 2011.

A casuística do estudo foi composta por um Grupo Controle (GC) de participantes sadios e dois grupos experimentais de sobreviventes.

O GC foi constituído por 30 participantes (15 homens e 15 mulheres), maiores de 18 anos, selecionados da população em geral. Foram incluídos somente participantes fisicamente saudáveis, com ausência de histórico de diagnóstico oncológico em qualquer momento da vida e recém-ingressos em curso superior. Com esses critérios, buscou-se constituir uma amostra de conveniência que permitisse uma comparação com indivíduos que, a princípio, seriam fisicamente sadios e com bom nível cultural.

Para os grupos experimentais, foram selecionados sobreviventes, maiores de 18 anos, de ambos os sexo, há pelo menos 5 anos em acompanhamento no Ambulatório Fora de Terapia do Instituto de Tratamento do Câncer Infantil (ITACI) do Serviço de Onco-Hematologia Pediátrica do HC-FMUSP. Os indivíduos foram divididos em grupo experimental composto por 30 sobreviventes de LLA (GLLA) e grupo experimental composto por 30 sobreviventes de TW (GTW).

A coleta de dados de todos os grupos foi realizada por meio da aplicação, por contato telefônico, do questionário SF-36, o qual foi validado para a língua portuguesa na forma presencial.^(12)^ Em estudo posterior de análise da viabilidade de uma forma de aplicação alternativa (via ligação telefônica), foi detectada a equidade das duas formas de apresentação − presencial e por telefone.^(13)^ O tempo médio de aplicação do questionário (via telefone) foi de 12,3 minutos. Os dados foram coletados de setembro de 2011 a agosto de 2013.

O participante foi previamente esclarecido quanto aos propósitos, ao procedimento e ao grau de risco da pesquisa à sua saúde. Mediante a sua concordância, foi firmado um Termo de Consentimento Livre e Esclarecido de participação na pesquisa.

Com o objetivo de enriquecer o estudo e estabelecer possíveis correlações relevantes nos resultados obtidos para os domínios do SF-36, além da coleta das respostas para as questões que constituem o instrumento utilizado, alguns dados clínicos e sociodemográficos atualizados foram selecionados por meio de consulta ao prontuário do paciente (idade no momento do diagnóstico e período de tempo fora de terapia) ou com o próprio participante, no momento do contato telefônico, antes da aplicação do SF-36 (estado civil, filhos, escolaridade, ocupação profissional, renda familiar bruta média).

Os dados obtidos a partir da aplicação do questionário SF-36 foram compilados de acordo com o critério de validação para a língua portuguesa.^(12)^ A renda familiar bruta dos participantes foi agrupada em duas faixas de salários mínimos (abaixo ou igual a 2,5 salários mínimos e acima de 2,5 salários mínimos), considerando como referência os valores obtidos no levantamento socioeconômico de 2011, realizado pelo Instituto Brasileiro de Opinião Pública e Estatística (IBOPE).^(14)^

A análise estatística dos resultados foi realizada utilizando o *software Statistical Package for the Social Sciences* (SPSS), por meio dos testes χ^2^, *t* independente e de análise de variância (ANOVA).

Foi considerado como nível de significância p=0,05, exceto uma análise em que foi examinada a significância p=0,10, pois, neste caso, foi evidenciada significância no teste ANOVA (p=0,05). Contudo, essa diferença nas comparações múltiplas de mínimos quadrados (LSMC - *least squares multiple comparison)* foi somente detectada para p=0,10.

## RESULTADOS

Na tabela 1, estão apresentadas as características sociodemográficas dos grupos experimentais e controle.

**Tabela 1:** Perfil sociodemográfico dos grupos de participantes

Características sociodemográficas		Grupos			Valor de p
		GLLA	GTW	GC	
		n (%)	n (%)	n (%)	
Idade (anos)	18-25	14 (46,6)	18 (60,0)	23 (76,6)	0,058^#^
	≥26	16 (53,4)	12 (40,0)	7 (23,4)	
Sexo	Feminino	15 (50,0)	15 (50,0)	15 (50,0)	
	Masculino	15 (50,0)	15 (50,0)	15 (50,0)	
Estado civil	Solteiro	25 (83,3)	21 (70,0)	27 (90,0)	0,131^#^
	Casado ou em união estável/divorciado/separado	5 (16,7)	9 (30,0)	3 (10,0)	
Filhos*	Sim	3 (10,0)	07 (23,4)	2 (6,6)	^##^
	Não	27 (90,0)	23 (76,6)	28 (93,4)	
Escolaridade	Ensino Fundamental incompleto até Ensino Médio completo	15 (50,0)	13 (43,3)	0 (0,0)	
	Superior incompleto	7 (23,4)	7 (23,4)	22 (73,4)	<0,001^###^
	Superior completo e pós-graduados	8 (26,6)	10 (33,4)	8 (26,6)	
Situação no mercado de trabalho	Empregado				^##^
	Cargos de chefia (supervisores, gerentes etc.)	4 (13,3)	6 (20,0)		
	Demais níveis hierárquicos	16 (53,4)	16 (53,4)	19 (63,4)	
	Autônomo	3 (10,0)	3 (10,0)	4 (13,3)	
	Desempregado	2 (6,6)	1 (3,3)		
	Atividades do lar	1 (3,4)	1 (3,3)		
	Estudante/não trabalha	4 (13,3)	03 (10,0)	7 (23,3)	
Proveniência demográfica (regiões)	Sudeste				^##^
	São Paulo	29 (96,7)	24 (80,0)	30 (100,0)	
	Minas Gerais	1 (3,3)	04 (13,4)		
	Rio de Janeiro		01 (3,3)		
	Sul				
	Paraná		01 (3,3)		
Renda familiar média bruta (SM)	≤2,5^$^	12 (40,0)	09 (30,0)		^##^
	>2,5^$$^	18 (60,0)	21 (70,0)	30 (100,0)	

^#^χ^2^ (p=0,05); ^##^χ^2^: aplicação inviável (baixa incidência de algumas categorias); ^###^χ^2^ (p=0,05); comparativo por partições GLLA *versus* GTW (p=0,083); GLLA *versus *GC (p<0,001); GTW *versus* GC (p<0,001); ^$^faixa de renda média bruta familiar referente ao levantamento socioeconômico de 2011 do IBOPE Mídia e da Associação Brasileira de Estudos Populacionais (ABEP),^(14)^ que abrange as classes C e D (Critério Brasil - 2013); ^$$^faixa de renda média bruta familiar referente ao levantamento socioeconômico de 2011 segundo IBOPE Mídia/ABEP,^(14)^ que abrange as classes A e B (Critério Brasil - 2013). GLLA: grupo experimental de sobreviventes de leucemia linfocítica aguda; GTW: grupo experimental de tumor de Wilms; GC: Grupo Controle; SM: salário mínimo.

Não houve evidência de diferença significativa quanto à idade dos participantes dos três grupos estudados (teste χ^2^ [2]=5,704; p=0,058), tendo o GC a maior concentração de participantes mais jovens (76,6% com até 25 anos) e o GLLA a maior concentração de sobreviventes com mais idade (53,4% acima de 26 anos).

A maioria dos participantes são solteiros, sem filhos e provenientes de São Paulo.

O GC apresentou grau de escolaridade superior em relação aos grupos experimentais. Isso ocorreu em razão do critério de inclusão no GC (teste χ^2^ [4]=27,02; p<0,001). No entanto, não houve diferença significante nos grupos experimentais entre si (teste χ^2^ [2]=0,365; p=0,833). Houve elevada concentração de sobreviventes graduados pelo menos até Ensino Médio (50,0% do GLLA e 43,3% do GTW) e considerável proporção de sobreviventes com Superior incompleto (23,4% do GLLA e 23,4% do GTW), completos e pós-graduados (26,6% do GLLA e 33,4% do GTW).

Quanto à ocupação, a maior parte dos participantes de todos os grupos era de empregados de níveis hierárquicos diversos, incluindo cargos de chefia. A renda familiar média esteve acima de 2,5 salários mínimos (Classes B e A conforme IBOPE-ABEP).^(14)^

Na tabela 2 estão apresentados os marcos clínicos dos participantes dos grupos experimentais.

**Tabela 2:** Características clínicas dos grupos experimentais

Características clínicas	GLLA	GTW	Valor de p
	Média (DP)	Média (DP)	
Idade ao diagnóstico (meses)	57,6 (37,2)	45,4 (25,8)	0,147
Período de tratamento (meses)	39,6 (18,0)	12,7 (7,9)	<0,001
Período fora de terapia (anos)	17,6 (4,7)	19,7 (4,6)	0,090

GLLA: grupo experimental de sobreviventes de leucemia linfocítica aguda; GTW: grupo experimental de sobreviventes de tumor de Wilms; DP: desvio padrão.

Quanto ao histórico terapêutico, não houve diferenças significativas quanto à idade no momento do diagnóstico (teste *t* independente, *t *(58)=1,472; p=0,147) e ao período de tempo fora de terapia para os sobreviventes analisados (teste *t* independente, *t* (56)=-1,725; p=0,090). Porém, no GTW, houve tendência à maior proporção de pacientes diagnosticados na faixa etária mais jovem, tendo sido 60% deles diagnosticados antes dos 52 meses de idade (média de 45,4 meses; desvio padrão − DP de 25,8) e, embora sem diferença significante, houve também nesse grupo maior proporção de sobreviventes há mais tempo fora de terapia: 64,3% dos sobreviventes há mais de 20 anos (média de 19,7 meses; DP de 4,6). Nota-se que o tratamento dispensado aos pacientes GLLA foi, em média, significativamente (teste *t* independente, *t* (58)=-7,37, p<0,001*) mais prolongado (média de 39,6 meses; DP de 18,0).

A tabela 3 apresenta uma síntese dos resultados significativos obtidos na análise estatística para os domínios do SF-36 em relação a idade, sexo e idade no momento do diagnóstico dos participantes do estudo.

**Tabela 3:** Análise estatística dos domínios do *Medical Outcomes Study 36-Item Short Form Health Survey* em relação aos fatores idade, sexo e idade do diagnóstico

Fatores/grupos	Média	DP	ANOVA	LSMC
			(Valor de p)	(Valor de p)
**Idade**	**Capacidade funcional (≤25/>26 anos)**			
GLLA	96,43/92,19	4,97/15,92		
GTW	93,61/90,42	7,24/10,33	0,038	
GC	94,78/85,71	10,92/20,90		0,072
**Sexo **	**Vitalidade (feminino/masculino)**			
GLLA	60,67/75,00	29,27/16,26		0,042
GTW	58,33/76,00	17,80/16,28	0,010	0,013
GC	63,00/63,00	19,35/11,77		
**Aspectos sociais (feminino/masculino)**				
GLLA	77,50/95,83	29,58/6,10		0,031
GTW	76,67/85,83	28,29/23,08	0,024	
GC	76,67/82,50	20,52/22,06		
**Aspectos emocionais (feminino/masculino)**				
GLLA	55,56/86,67	46,58/24,56		0,040*
GTW	55,56/71,11	46,58/45,19	0,038	0,040*
GC	82,22/60,00	33,01/44,01		
**Saúde mental (feminino/masculino)**				
GLLA	63,47/79,47	23,07/16,20		0,012
GTW	65,60/73,60	19,64/18,50	0,028	
GC	73,87/74,13	11,80/10,57		
**Idade do diagnóstico**	**Capacidade funcional (≤52/>53 meses)**			
GLLA	90,94/97,86	15,08/4,69		0,041**
GTW	94,17/89,58	6,70/10,54	0,033	

*GLLA masculino/GTW feminino; **GLLA >53/GTW >53; DP: desvio padrão; ANOVA: análise de variância; LSMC: comparações múltiplas de mínimos quadrados; GLLA: grupo experimental de sobreviventes de leucemia linfocítica aguda; GTW: grupo experimental de sobreviventes de tumor de Wilms; GC: Grupo Controle.

Quanto à idade dos participantes, foi evidenciada diferença significativa [ANOVA, (F [1,84]=4,438; p=0,038*)] para o domínio Capacidade funcional. Essa diferença só foi detectada quando se adotou o nível de significância (p) de 0,10; os participantes do GC mais jovens apresentaram maiores respostas para esse domínio (p=0,072*).

Também houve diferença significativa [ANOVA, (F [1,56]=4,780; p=0,033*)] para o domínio Capacidade funcional quando se considerou o fator idade no momento do diagnóstico. Dentre os sobreviventes que tiveram seus diagnósticos mais tardiamente (acima de 53 meses de idade), os do GLLA apresentaram valores de respostas significativamente maiores (p=0,041*).

Quanto ao sexo, foram encontradas diferenças significativas somente entre os sobreviventes, especificamente para os domínios Vitalidade [ANOVA, (F [1,84]=6,933; p=0,010*)], Aspectos sociais [ANOVA, (F [1,84]=5,286; p=0,024*)], Aspectos emocionais [ANOVA, (F [2,84]=3,382; p=0,038*)] e Saúde mental [ANOVA, (F [1,84] =4,980; p=0,028*)]. Os sobreviventes do sexo masculino dos grupos GLLA (p=0,044) e GTW (p=0,013) apresentaram valores maiores para as respostas do domínio Vitalidade. Os sobreviventes do sexo masculino do GLLA (p=0,031) apresentaram valores maiores para as respostas do domínio Aspectos sociais. Entre os participantes de um mesmo grupo, somente no GLLA (p=0,040) os sobreviventes apresentaram resultados maiores para o domínio Aspectos emocionais. Contudo, para participantes de grupos diferentes, notou-se que os sobreviventes do GLLA apresentaram respostas mais elevadas (p=0,040) para o domínio Aspectos emocionais, quando comparados aos do GTW. Os sobreviventes do GLLA apresentaram maiores valores para as respostas do domínio Saúde mental (p=0,012*).

A tabela 4 apresenta a percepção dos participantes da própria saúde (questão 1 do SF-36, cujas respostas possíveis são boa, muito boa e excelente) em relação aos domínios do SF-36.

**Tabela 4:** Análise estatística do fator percepção da própria da saúde do *Medical Outcomes Study 36 – Item Short Form Health Survey*

Fatores grupos	Média	DP	ANOVA (Valor de p)	LSMC (Valor de p)
**Percepção da saúde**	**Capacidade funcional (boa/muito boa/excelente)**			
GLLA	85,00/95,00/98,67	19,46/7,07/2,97		0,006^§^; 0,025^§§^; 0,021^§§§^
GTW	87,73/92,50/96,82	9,84/8,02/5,13	0,002	0,015^£^; 0,048^££^;
GC	90,36/91,67/98,57	15,75/16,20/2,44		0,048^£££^
**Aspectos físicos (boa/muito boa/excelente)**				
GLLA	78,13/100,0/100,0	33,91/0,00/0,00		0,019^#^
GTW	84,09/87,50/97,73	35,83/26,73/7,54	0,0250	
GC	78,57/69,44/96,43	30,79/41,04/9,45		0,026^##^; 0,005^###^; 0,015^#$^; 0,039^#$$^
**Dor (boa/muito boa/excelente)**				
GLLA	63,00/75,57/83,13	24,09/19,60/18,32		0,024^&^;0,021^&&^; 0,012^&&&^
GTW	65,18/70,38/84,73	16,98/22,80/20,16	0,0021	0,026^¢^; 0,024^¢¢^; 0,014^¢¢¢^
GC	73,29/79,00/89,43	20,48/24,14/10,50		
**Estado geral da saúde (boa/muito boa/excelente)**				
GLLA	67,63/81,57/94,33	25,56/15,08/8,42		0,0004q; 0,003qq; 0,003qqq
GTW	62,45/72,00/90,45	21,03/12,82/11,28	<0,0001	0,018w; 0,014e; <0,0001r; 0,0001t; 0,0002y; 0,002p; 0,018l; 0,013k
GC	57,00/80,89/93,57	24,02/7,41/7,48		0,001j; 0,043h; 0,001g; <0,0001d; 0,0001s; 0,0001a
**Vitalidade (boa/muito boa/excelente)**				
GLLA	46,25/65,00/80,67	27,48/14,72/17,71		0,014z; 0,011c; <0,0001v; 0,006b
GTW	62,73/69,38/70,00	24,12/12,94/17,75	0,0032	
GC	58,21/69,44/64,29	18,87/11,58/11,34		0,001n
**Saúde mental (boa/muito boa/excelente)**				
GLLA	56,50/66,29/81,87	27,91/18,60/11,70		0,046m; 0,023aa; 0,001^€^; 0,0247∞; 0,047Ω
GTW	66,18/72,50/70,91	21,34/14,88/20,87	0,0305	0,022^¶^
GC	71,71/75,56/76,57	11,26/11,39/10,69		

^§^boa GLLA/excelente GLLA; ^§§^boa GLLA/excelente GTW; ^§§§^boa GLLA/excelente GC; ^£^boa GTW/excelente GLLA; ^££^boa GTW/excelente GC; ^£££^boa GC/excelente GLLA; ^#^muito boa GLLA/muito boa GC; ^##^boa GC/excelente GLLA; ^###^muito boa GC/excelente GLLA; ^#$^muito boa GC/excelente GTW; ^#$$^muito boa GC/excelente GC; ^&^boa GLLA/excelente GLLA; ^&&^boa GLLA/excelente GTW; ^&&&^boa GLLA/excelente GC; ^¢^boa GTW/excelente GLLA; ^¢¢^boa GTW/excelente GTW; ^¢¢¢^boa GTW/excelente GC; qboa GLLA/excelente GLLA; qqboa GLLA/excelente GTW; qqqboa GLLA/excelente GC; wboa GTW/muito boa GLLA; eboa GTW/muito boa GC; rboa GTW/excelente GLLA; tboa GTW/excelente GTW; yboa GTW/excelente GC; pmuito boa GTW/excelente GLLA; lmuito boa GTW/excelente GTW; kmuito boa GTW/excelente GC; jboa GC/muito boa GLLA; hboa GC/muito boa GTW; gboa GC/ muito boa GC; dboa GC/excelente GLLA; sboa GC/excelente GTW; aboa GC/excelente GC; zboa GLLA/muito boa GTW; cboa GLLA/muito boa GC; vboa GLLA/excelente GLLA; bboa GLLA/excelente GTW; nboa GC/excelente GLLA; mboa GLLA/boa GC; aaboa GLLA/excelente GLLA; ^€^boa GLLA/muito boa GC; ∞boa GLLA/excelente GC; Ωmuito boa GLLA/excelente GLLA; ^¶^boa GTW/excelente GLLA. DP: desvio padrão; ANOVA: análise de variância; LSMC: comparações múltiplas de mínimos quadrados; GLLA: grupo experimental de sobreviventes de leucemia linfocítica aguda; GTW: grupo experimental de sobreviventes de tumor de Wilms; GC: Grupo Controle.

Observou-se diferença estatística para todos os grupos quanto à percepção da própria saúde para os domínios analisados pelo SF-36, exceto Aspectos sociais e Aspectos emocionais. Nota-se que as diferenças significativas encontradas se estabelecem em alguns casos internamente, entre participantes de um mesmo grupo, mas também entre participantes de grupos diferentes. A relação completa das relações significativas foi apresentada na legenda da tabela 4.

## DISCUSSÃO

Ser acometido por um câncer e sobreviver após a remissão podem ser momentos vivenciados com dor e sofrimento pelos pacientes e seus familiares. É destacado, nas análises sobre a terapêutica, que os avanços alcançados no tratamento e os esforços para a melhoria da QVRS não evitam situações disruptivas estabelecidas pelo impacto do diagnóstico, pelo tratamento e por suas implicações.^(15)^ De acordo com essa perspectiva de comprometimento da QVRS na sobrevivência, este estudo encontrou resultados a serem discutidos.

Quanto à percepção da própria saúde, houve diferenças significativas para todos os domínios, exceto para Aspectos sociais e Aspectos emocionais. No entanto, notou-se que as diferenças encontradas para GLLA, GTW e GC se estabeleceram em torno da percepção de saúde boa, muito boa e excelente, evidenciando que todos os participantes não pareceram denotar percepção negativa de sua própria saúde.

Nos grupos experimentais dos sobreviventes, a idade não apresentou significativa influência nos resultados para todos os domínios do SF-36 e, portanto, nem na QVRS desses sobreviventes.

Diversos estudos trataram da sobrevida da LLA e de seus possíveis comprometimentos. São relevantes, para analisar os resultados obtidos por este estudo, os trabalhos de Robison,^(16)^ Ortolan^(17)^ e Zebrack et al.^(18)^ O estudo de Robison^(16)^ abordou a sobrevida de LLA em estudo coorte. Apesar de não estabelecer uma análise de QVRS, o trabalho analisou os efeitos tardios na sobrevida de LLA, com destaque para os comprometimentos psíquicos e sociais nos sobreviventes de ambos os sexos − condição não observada no presente estudo. Os melhores escores de determinados aspectos da QVRS dos sobreviventes do sexo masculino em comparação às sobreviventes de LLA obtidos neste estudo corroboram resultados previamente divulgados por Ortolan,^(17)^ especificamente para os domínios Vitalidade, Aspectos sociais e Saúde mental. Zebrack et al.^(18)^ analisaram particularmente o funcionamento psicossocial futuro do sobrevivente de LLA, encontrando que os sobreviventes eram mais propensos a relatar aspectos da sintomatologia depressiva e somática do que os controles − as mulheres foram mais propensas a indicar sintomas relacionados à depressão e angústias somáticas que os homens. Na amostra analisada por este estudo, comprometimentos psíquicos também foram evidenciados com diferenças significativamente menores nos domínios Saúde mental e Aspectos emocionais do SF-36 das sobreviventes GLLA.

É importante ressaltar que, apesar das diferenças acima descritas, não foram detectados valores médios abaixo de 50 (valor médio da escala) para nenhum dos domínios avaliados, em ambos os sexos.

Das variáveis clínicas analisadas, dos sobreviventes com diagnóstico tardio (acima de 53 meses), somente o domínio Capacidade funcional mostrou resultados significativamente maiores para o GLLA, diversamente do obtido por Zebrack et al.,^(18)^ que destacaram serem a idade no momento do diagnóstico, o tempo de diagnóstico e a duração do tratamento preditivos de escores sintomáticos para depressão ou desconfortos somáticos_._^(18)^

Por outro lado, há poucas análises sobre a QVRS dos dois grupos de comprometimento neoplásico (hematológico e sólido, especificamente LLA e TW), comparando os quadros de sobrevida entre si e particularmente para a faixa etária analisada neste estudo, como, por exemplo Mackie et al.,^(9)^ Stuber e Shemesh.^(10)^

Mackie et al.^(9)^ analisaram transtornos psiquiátricos, desempenho interpessoal/social e capacidade intelectual de 102 pacientes (19 a 30 anos) sobreviventes de LLA e TW acometidos na infância e sem recaídas durante 5 anos comparativamente a um GC de sujeitos saudáveis. Quanto aos transtornos psiquiátricos, não houve diferenças significativas entre os grupos analisados. *Déficits* dos itens relacionamento amor/sexo e amizade, encontrados entre os sobreviventes, foram associados a situações de tratamento mais recente.^(9)^

O estudo realizado por Mackie et al.^(9)^ se aproxima deste trabalho quanto à faixa etária e ao acometimento neoplásico, mas focaliza a análise da sobrevida especificamente quanto aos transtornos psiquiátricos e ao desempenho social e intelectual, apontando *déficits* não detectados por este estudo e não abordando outros aspectos da sobrevida, como, por exemplo, o físico.

Stuber e Shemesh^(10)^ analisaram o transtorno de estresse pós-traumático em crianças e adolescentes acometidos por diversas neoplasias, e em seus pais. Apesar de incluir sobreviventes mais jovens (8 a 20 anos), o trabalho de Stuber e Shemesh comparou os acometimentos estudados por este trabalho (309 sobreviventes, sendo 38% deles com de LLA e 10% de TW) em relação ao GC saudável pareados pela idade. Stuber e Shemesh^(10)^ também não evidenciaram diferença significativa entre os escores de sintomas classificáveis entre grave e moderado para o transtorno, quando comparados os grupos sobreviventes e controle saudável, tal qual observado no presente estudo, quanto à ausência de diferença significativa de transtornos psíquicos dos sobreviventes em relação ao controle.

Koch et al.^(11)^ analisaram a QVRS de sobreviventes e não também encontraram sérios comprometimentos dos indivíduos sobreviventes nos mesmos acometimentos estudados por este trabalho em relação a GC sadios. Os autores trataram de aspectos psicossociais pela possibilidade de sair da casa dos pais dos sobreviventes. Sobreviventes de tumores hematológicos e sólidos não diferiram significativamente em relação ao GC quanto à variável sair da casa dos pais. Concluíram que, para o sobrevivente e seus pais, ter tido câncer na infância e o tipo de tratamento exigido (cirurgia e/ou quimioterapia) não interferiram significativamente no processo psicossocial geral e nem sobre ter independência social na fase adulta.^(11)^ Contudo, ao abordarem a sobrevida de pacientes adultos acometidos por câncer hematológico e tumor sólido, os autores analisaram conjuntamente as leucemias/linfomas e todos os tumores sólidos agrupados, impedindo uma especificidade de análise para cada acometimento, fato que propiciaria uma comparação mais rigorosa em relação aos dados obtidos por este estudo.

Quanto a aplicação do SF-36 por telefone, preconizada neste estudo, Zebrack e Landier^(19)^ ressaltaram a importância do acompanhamento de suporte para sobreviventes de câncer, incluindo serviços *on-line.* Meadows^(7)^ analisou a detecção de situações de estresse pós-traumático e a relutância do sobrevivente a retornar ao local de tratamento, que é associado à condição traumatizante. Dessa forma, o diferencial proposto pode ser considerado mais um recurso de ajuda no *déficit* de aproximação e obtenção de dados para o seguimento clínico, favorecendo a obtenção de informações para a investigação científica, acompanhamento de sua condição clínica e intervenção, se for necessário.^(7,19)^

É relevante ressaltar que há poucas análises sobre a qualidade de vida relacionada à saúde comparando sobreviventes de leucemia linfocítica aguda e tumor de Wilms. Assim, este estudo buscou contribuir para a construção de conhecimento sobre o tema, o acúmulo de evidências e o aprimoramento dos processos terapêuticos.

Maior número de análises podem detectar particularidades, enriquecer os conhecimentos prévios e dar margem a estudos futuros, como se buscou nesta pesquisa para os sobreviventes de LLA e TW.

## CONCLUSÃO

Sobreviventes do sexo masculinos tiveram resultados melhores em relação às sobreviventes e aos participantes do Grupo Controle. Especificamente, nos grupos experimentais de sobreviventes de leucemia linfocítica aguda e de tumor de Wilms para o domínio Vitalidade, e no grupo experimental de sobreviventes de leucemia linfocítica aguda para Aspectos sociais, Saúde mental e Aspectos emocionais, sendo para esse último aspecto também detectada diferença em relação às sobreviventes do grupo experimental de sobreviventes de tumor de Wilm.

Somente dentre os sobreviventes com diagnóstico tardio (acima dos 53 meses de idade), houve diferenças significantes de qualidade de vida, onde o grupo experimental de sobreviventes de leucemia linfocítica aguda apresentou melhores resultados quanto ao domínio Capacidade funcional.

Em relação à percepção da própria saúde, houve diferença para todos os domínios, exceto para os Aspectos sociais e Aspectos emocionais. No entanto, as diferenças encontradas estiveram relacionadas a percepções positivas (boa, muito boa e excelente) da própria saúde dos sobreviventes e controle sadio.

Pode-se inferir, para o período de realização e para amostra analisada, que os sobreviventes analisados não apresentaram evidências de comprometimento de qualidade de vida relacionada à saúde para os aspectos analisados pelo instrumento utilizado, bem como se comparados ao Grupo Controle de participantes sadios.

Os resultados encontrados são passíveis de confirmação por estudos futuros, em outras amostras de participantes, por outros centros de pesquisa e com outros instrumentos de avaliação da qualidade de vida relacionada à saúde.

Cabe acrescentar que a utilização do *Medical Outcomes Study 36 *– *Item Short Form Health Survey* via telefone viabilizou o acesso e a avaliação da qualidade de vida de sobreviventes, sob acompanhamento ambulatorial.
